# One new genus *Najas* (Hydrocharitaceae) and four new vascular plant records for Kosovo: *Lycopodium*, *Najas* and two *Cyperus* species

**DOI:** 10.3897/BDJ.14.e181774

**Published:** 2026-01-19

**Authors:** Fadil Millaku, Elez Krasniqi, Pajtim Bytyçi, Naim Berisha

**Affiliations:** 1 Department of Biology, Faculty of Mathematics and Natural Sciences, University of Prishtina, Prishtina, Kosovo Department of Biology, Faculty of Mathematics and Natural Sciences, University of Prishtina Prishtina Kosovo; 2 UBT – Higher Education Institution, Prishtina, Kosovo, Prishtina, Kosovo UBT – Higher Education Institution, Prishtina, Kosovo Prishtina Kosovo

**Keywords:** Balkan flora, floristic inventory, aquatic macrophytes, high-mountain habitats

## Abstract

**Background:**

Targeted field surveys, during the 2025 vegetative period, across a range of natural habitats revealed four vascular plant taxa previously unconfirmed for the flora of Kosovo: Lycopodium annotinum subsp. annotinum, *Najas marina*, *Cyperus fuscus* and *C. rotundus*. Specimens were collected from upper-montane spruce forests, in lake and littoral zones and serpentine foothill streams and were identified using standard regional floras; all vouchers are deposited in the Herbarium of the University of Prishtina (UPH). The discovery of L. annotinum subsp. annotinum adds a new boreal-montane element to the national Lycopodiaceae, while *N. marina* represents the first documented member of the genus *Najas* in the country. Likewise, *C. fuscus* and *C. rotundus* are the first confirmed representatives of *Cyperus*, demonstrating that cyperaceous diversity in Kosovo has been significantly underestimated. These findings highlight the ecological and biogeographical complexity of the region, emphasise the importance of wetlands, aquatic systems, upper-montane coniferous forests and serpentine landscapes for harbouring overlooked taxa and underscore the need for continued systematic floristic surveys to refine the national inventory and support future conservation and biodiversity research.

**New information:**

Four new species are being reported for the flora of Kosovo: Lycopodium annotinum subsp. annotinum, *Najas marina*, *Cyperus fuscus* and *C. rotundus*.

## Introduction

The Balkan Peninsula is widely recognised as one of Europe’s major centres of plant diversity and endemism, shaped by its complex geological history, heterogeneous topography and contrasting climatic regimes (Willner et al. 2009, Nieto Feliner 2014, Niketić et al. 2021, Španiel and Rešetnik 2022). The region’s mosaic of mountain systems, extending from the Dinarides and Albanian Alps to the Sharr‐Pindus system and the Balkan Range, provides a wide range of ecological niches that have supported both ancient Tertiary lineages and more recent postglacial flora (Đurović et al. 2021, Španiel and Rešetnik 2022). Many species in the central and southern Balkans survived the Pleistocene climatic oscillations in multiple microrefugia, contributing to today’s exceptional floristic richness and genetic structure (Caković and Frajman 2020, Đurović et al. 2021, Berisha et al. 2025c).

Several recent studies continue to emphasise the importance of the Balkans as a hotspot for narrowly distributed taxa, relict species and habitat specialists, including chasmophytes (Stešević et al. 2023), subalpine–alpine grassland elements (Tzonev et al. 2025), edaphic endemics on serpentine or calcareous substrates (Kuzmanović et al. 2017) and numerous lineages exhibiting amphi-Adriatic or Balkan-Apennine disjunctions (Stevanoski et al. 2020). Despite more than a century of floristic investigation, the Balkan Peninsula remains far from fully documented. New national records continue to be reported, especially from mountain and wetland habitats (Sabovljevic et al. 2024), while range extensions and overlooked populations are still being identified in several taxonomic groups (Krasniqi et al. 2020, Berisha et al. 2020, Berisha et al. 2025b). In addition, the region has recently yielded newly-described species and even new genera, demonstrating that its floristic diversity is still incompletely explored (Millaku and Elezaj 2015, Bogdanović et al. 2024, Surina et al. 2025). Together, these findings underline that large parts of the Balkans, despite longstanding botanical interest, remain insufficiently surveyed and continue to offer important floristic insights.

Kosovo is located at the intersection of the Dinaric, Scardo–Pindic and Balkan biogeographical provinces, thereby reflecting a regional pattern of outstanding, yet incompletely documented plant diversity. Its biogeographical position places the country within a transitional zone where Alpine, Continental and sub-Mediterranean influences naturally overlap (Rexhepi 1997, Berisha et al. 2025a). Although the country’s vascular flora has been intensively studied in the last two decades, numerous taxonomic groups, wetland systems and upland habitats remain poorly explored. Many species with cryptic morphology, ephemeral phenology or habitat specialisation, have historically been overlooked or reported with uncertainty (Millaku et al. 2025).

Across the Balkans, including Kosovo and its neighbouring countries, fieldwork continues to reveal species that had long gone unrecorded, even in relatively accessible habitats. These discoveries indicate that many wetlands, forest openings and high-elevation areas remain insufficiently explored and that ongoing surveys are essential for improving national floristic inventories. In Kosovo, such efforts have already added several verified records, helping to clarify the country’s plant diversity and its place within the wider Balkan flora.

During the 2025 vegetative period, intensive field surveys across a range of habitats resulted in the confirmed presence of four vascular plant taxa previously unreported and unconfirmed for the flora of Kosovo: Lycopodium annotinum L. subsp. annotinum, *Najas marina* L., *Cyperus fuscus* L. and *Cyperus rotundus* L. These findings are particularly relevant given that all four genera contain taxa of ecological importance and conservation significance. Lycophytes are often sensitive indicators of forest and soil conditions (García Criado et al. 2017); *Najas* species reflect freshwater ecosystem health (Wingfield et al. 2006); and *Cyperus* species contribute to wetland structure and hydrological function (Mishra et al. 2015). Accurate documentation of their national occurrence is thus relevant not only for floristics, but also for habitat assessment, biogeographical analyses and conservation planning.

Given Kosovo’s position in the centre of the Balkan biodiversity hotspot, documenting new species occurrences contributes fundamentally to refining regional distribution maps, identifying cross-border floristic patterns and detecting potential range shifts under current environmental changes. It also strengthens the country’s integration into broader Balkan and European floristic syntheses.

The aims of this study were: (***i***) to document and formally report four vascular plant taxa as new records for the flora of Kosovo; (***ii***) to provide verified ecological and distributional information for each taxon, supported by field observations and herbarium material; (***iii***) to clarify their national occurrence within the biogeographical context of Kosovo; and (***iv***) to underline the ecological and conservation relevance of these new findings.

## Materials and methods

This study is part of our continuous, long-term botanical work conducted under several national-scale projects focused on documenting the vascular flora and natural habitats of Kosovo. During these activities, we visited a wide range of natural habitats across the country throughout the 2025 vegetative period. Surveys covered wetlands, riparian and aquatic habitats, wet meadows, marshy forest openings, coniferous and broadleaf forests, as well as subalpine and high-elevation grasslands. These field visits were carried out during periods when the target vegetation was most readily identifiable.

Specimens were collected using standard botanical procedures, ensuring that well-developed individuals were sampled. For each taxon, notes were taken on habitat characteristics, associated plant species, microtopography and general ecological conditions. All specimens were subsequently examined in detail using regional floristic literature, including the main Balkan floras: flora of Albania (Paparisto et al. 1988, Qosja et al. 1992, Qosja et al. 1996, Vangjeli et al. 2000, Pils 2016), flora of Serbia (Josifović 1970a, Josifović 1970b, Josifović 1972a, Josifović 1972b, Josifović 1973, Josifović 1974, Josifović 1975, Josifović 1976, Josifović 1977, Sarić and Diklić 1986, Stevanović 1992, Stevanović 2012) and Mountain flora of Greece (Strid 1986, Strid and Tan 1991). Nomenclature follows current usage in Euro+Med PlantBase (Euro+Med 2006 + continuously updated 2025).

Voucher specimens were prepared and deposited in the Herbarium of the University of Prishtina (UPH). Geographic coordinates and elevations were recorded in the field using hand-held GPS devices. To confirm the novelty of each finding, all records were carefully checked against the existing literature and available herbarium materials for Kosovo. Only taxa without any previously confirmed national records are presented here.

## Data resources

During the 2025 vegetation season, four vascular plant taxa were confirmed as new records for the flora of Kosovo. Each taxon is presented below with verified locality information, habitat descriptions and voucher specimens deposited in the Herbarium of the University of Prishtina (UPH).

## Taxon treatments

### Lycopodium annotinumannotinum

Linnaeus 1753

CDAEBC04-3D5E-5717-8F07-7F284F63057B

urn:lsid:ipni.org:names:321282-2

= Spinulum annotinum (L.) A. Haines subsp. annotinum

#### Materials

- **Type status:** Other material. **Occurrence:** catalogNumber: UPH-00002335; occurrenceRemarks: inside of Picea abies forest; recordNumber: 21; recordedBy: Naim Berisha; individualCount: 30; occurrenceStatus: Present; occurrenceID: AA0930AE-8338-5A0A-85D5-695727FC1EC3; **Taxon:** taxonID: urn:lsid:ipni.org:names:304983-2; scientificNameID: urn:lsid:ipni.org:names:304983-2; acceptedNameUsage: Lycopodium annotinum L. subsp. annotinum; parentNameUsage: Lycopodiaceae; originalNameUsage: Spinulum annotinum (L.) A. Haines subsp. annotinum; nameAccordingTo: Christenhusz, M. J. M. & Raab-Straube, E. von 2013+: Lycopodiophytina. – In: Euro+Med Plantbase - the information resource for Euro-Mediterranean plant diversity. Published at https://europlusmed.org/.; kingdom: Plantae ; phylum: Tracheophyta ; class: Lycopodiopsida ; order: Lycopodiales ; family: Lycopodiaceae ; genus: Lycopodium ; specificEpithet: annotinum; taxonRank: subspecies; scientificNameAuthorship: (L.) A. Haines; nomenclaturalCode: IPNI; taxonomicStatus: Accepted; **Location:** higherGeographyID: SE Europe; higherGeography: SE Europe; continent: Europe; country: Kosovo; countryCode: XK; stateProvince: Peja; county: Istog; municipality: Istog; locality: Albanian Alps of Kosovo - Mokna Mt.; verbatimLocality: Mokna; verbatimElevation: 1551 m; locationRemarks: Albanian Alps of Kosovo - Mokna Mt., inside of Picea abies forest: 42.908297°N, 20.547309°E - at 1551 m a.s.l. 16 Nov. 2025. Leg.: Naim Berisha & Fadil Millaku.; verbatimCoordinates: 42.908297°N, 20.547309°E; coordinatePrecision: 2; georeferenceProtocol: GPS; **Identification:** identifiedBy: Naim Berisha; dateIdentified: 2025; identificationReferences: Tutin, T. G., Heywood, V. H., Burges, N. A., Moore, D. M., Valentine, D. H., Walters, S. M., Webb, D. A. (1964): Flora Europaea - Volume 1. Lycopodiaceae to Platanaceae. Cambridge University Press.; **Event:** eventDate: 16/11/2025; habitat: Picea abies forest, moist.

#### Taxon discussion

This taxon has not previously been reported for the flora of Kosovo; thus, it represents the first verified record of the species in Kosovo. The present record is based on field-collected material that was taxonomically verified using current regional floras and compared with herbarium material from UPH. Its occurrence extends the known distribution range of the species in the central Balkans and contributes to completing the floristic knowledge of Kosovo. No earlier confirmed records exist in the national or regional literature.

#### Notes

This taxon was recorded in Mokna Mt.; it is thriving in moist, shaded microhabitats within Norway spruce (*Picea abies* (L.) H. Karst.) forest; growing near wet rocks with abundant bryophytes (Fig. 1a). Concerning its chorology, it is a Boreal–montane Eurasian species, which, in the Balkans, is mainly confined to high-elevation coniferous forests. Its presence suggests suitable microrefugial conditions in upper-montane coniferous forests.

### Najas marina

Linnaeus 1753

301F5AA1-7A40-509B-80B7-5B3F581CF548

urn:lsid:ipni.org:names:321282-2

#### Materials

- **Type status:** Other material. **Occurrence:** catalogNumber: UPH-00002336; occurrenceRemarks: lake shallow waters; recordNumber: 1; recordedBy: Fadil Millaku; individualCount: 20; occurrenceStatus: Present; occurrenceID: 65FE973E-B976-5769-A4A9-4BC807541539; **Taxon:** taxonID: urn:lsid:ipni.org:names:321282-2; scientificNameID: urn:lsid:ipni.org:names:321282-2; acceptedNameUsage: Najas marina L.; parentNameUsage: Hydrocharitaceae; nameAccordingTo: Uotila, P. 2009+: Hydrocharitaceae. – In: Euro+Med Plantbase - the information resource for Euro-Mediterranean plant diversity. Published at https://europlusmed.org/; kingdom: Plantae; phylum: Tracheophyta; class: Magnoliopsida; order: Alismatales; family: Hydrocharitaceae; genus: Najas; specificEpithet: marina; taxonRank: species; scientificNameAuthorship: L.; nomenclaturalCode: IPNI; taxonomicStatus: Accepted; **Location:** higherGeographyID: SE Europe; higherGeography: SE Europe; continent: Europe; country: Kosovo; countryCode: XK; stateProvince: Gjakovë; county: Gjakovë; municipality: Gjakovë; locality: Radoniq lake; verbatimLocality: Radoniq lake; verbatimElevation: 462 m; locationRemarks: Gjakova district – Radoniq Lake, in its shallow waters: 42.479880°N, 20.408592°E, 462 m a.s.l., 12 Aug. 2025. Leg.: Fadil Millaku; verbatimCoordinates: 42.479880°N, 20.408592°E; coordinatePrecision: 2; georeferenceProtocol: GPS; **Identification:** identifiedBy: Fadil Millaku; dateIdentified: 2025; identificationReferences: Simpson, D.A. (1980): Najas. — In: Tutin, T.G. et al. (eds.), Flora Europaea, Vol. 5, Alismataceae to Orchidaceae: 6–8. Cambridge University Press, Cambridge.; **Event:** eventDate: 12/08/2025; habitat: Shallow lake waters.

#### Taxon discussion

This taxon has not previously been reported for the flora of Kosovo, thus representing its first verified record in Kosovo. The present record is based on field-collected material that was taxonomically verified using current regional floras and compared with herbarium material from UPH. Its occurrence extends the known distribution range of the species in the central Balkans and contributes to completing the floristic knowledge of Kosovo. No earlier confirmed records exist in the national or regional literature.

#### Notes

The species was recorded along the shores of Radoniq Lake, where it was growing as a submerged macrophyte in shallow, calm waters near the Lake margin (Fig. 1b). Ecologically, the species occupies nutrient-rich standing waters and is often associated with slow-flowing or stagnant aquatic habitats. In a broader biogeographical context, *Najas marina* is a widespread Eurasian hydrophyte, occurring in lowland to montane freshwater systems. The documented presence of this species in Kosovo adds an important aquatic element to the national flora. Its presence indicates suitable ecological conditions for submerged vegetation in the periodically inundated drawdown zone of Radoniq Lake.

### Cyperus fuscus

Linnaeus 1753

CD30EC09-71FC-530A-848E-E2C1A12E2DA7

urn:lsid:ipni.org:names:330929-2

= *Cyperus virescens* Hoffm.

#### Materials

- **Type status:** Other material. **Occurrence:** catalogNumber: UPH-00002333; occurrenceRemarks: along a small seasonal stream below pastures; recordNumber: 5; recordedBy: Naim Berisha; individualCount: 16; occurrenceStatus: Present; occurrenceID: 44A7F482-960D-57FA-B906-6F3D06F0B520; **Taxon:** taxonID: urn:lsid:ipni.org:names:330929-2; scientificNameID: urn:lsid:ipni.org:names:330929-2; acceptedNameUsage: Cyperus fuscus L.; parentNameUsage: Cyperaceae; originalNameUsage: Cyperus virescens Hoffm.; nameAccordingTo: Jiménez-Mejías, P. & Luceño, M. 2011+: Cyperaceae. – In: Euro+Med Plantbase - the information resource for Euro-Mediterranean plant diversity. Published at https://europlusmed.org/.; kingdom: Plantae; phylum: Tracheophyta; class: Magnoliopsida; order: Poales ; family: Cyperaceae ; genus: Cyperus ; specificEpithet: fuscus; taxonRank: species; scientificNameAuthorship: L.; nomenclaturalCode: IPNI; taxonomicStatus: Accepted; **Location:** higherGeographyID: SE Europe; higherGeography: SE Europe; continent: Europe; country: Kosovo; countryCode: XK; stateProvince: Prishtina; county: Gllogoc; municipality: Gllogoc; locality: Golesh Mt.; verbatimLocality: Golesh Mt.; verbatimElevation: 743 m; locationRemarks: Golesh Mt. – Mirenë, along a small seasonal stream below pastures, serpentine substrate: 42.563104°N, 20.967532°E, 743 m a.s.l., 05 Oct. 2025. Leg.: Naim Berisha.; verbatimCoordinates: 42.563104°N, 20.967532°E; coordinatePrecision: 2; georeferenceProtocol: GPS; **Identification:** identifiedBy: Naim Berisha; dateIdentified: 2025; identificationReferences: Kukkonen, I. (1980): Cyperus. — In: Tutin, T.G., Heywood, V.H., Burges, N.A., Moore, D.M., Valentine, D.H., Walters, S.M. & Webb, D.A. (eds.), Flora Europaea, Vol. 5: 282–292. Cambridge University Press, Cambridge.; **Event:** eventDate: 5/10/2025; habitat: seasonal stream below pastures, serpentine substr.

#### Taxon discussion

This taxon has not previously been reported for the flora of Kosovo. The present record is based on field-collected material that was taxonomically verified using current regional floras and compared with herbarium material from UPH. Its occurrence extends the known distribution range of the species in the central Balkans and contributes to completing the floristic knowledge of Kosovo. No earlier confirmed records exist in the national or regional literature.

#### Notes

The species was recorded on Golesh Mt. (Mirenë), being found along a small seasonal stream flowing beneath open pastures on serpentine substrate (Fig. 1c). The species typically occupies moist, intermittently flooded microhabitats such as muddy stream margins, ephemeral wet depressions and disturbed wetland edges. Biogeographically, *Cyperus fuscus* is a widespread European and western Eurasian sedge, although often overlooked due to its small stature and short phenological window. This collection represents the first verified record of the species for Kosovo, demonstrating that serpentine foothill habitats and small temporary watercourses may support a richer sedge flora than previously recognised.

### Cyperus rotundus

Linnaeus 1753

8AA18746-483F-529C-935E-790EA8042184

urn:lsid:ipni.org:names:305797-1

= *Chlorocyperus rotundus* (L.) Palla
= *Cyperus tuberosus* Rottb.

#### Materials

- **Type status:** Other material. **Occurrence:** catalogNumber: UPH-00002334; occurrenceRemarks: littoral zone, moist sandy–loamy shoreline; recordNumber: 7; recordedBy: Fadil Millaku; individualCount: 10; occurrenceStatus: Present; occurrenceID: E3E1056D-70A1-521A-AB78-C0CAD6FF877D; **Taxon:** taxonID: urn:lsid:ipni.org:names:305796-1; scientificNameID: urn:lsid:ipni.org:names:305796-1; acceptedNameUsage: Cyperus rotundus L.; parentNameUsage: Cyperaceae; originalNameUsage: Cyperus tuberosus Rottb.; nameAccordingTo: Jiménez-Mejías, P. & Luceño, M. 2011+: Cyperaceae. – In: Euro+Med Plantbase - the information resource for Euro-Mediterranean plant diversity. Published at https://europlusmed.org/.; kingdom: Plantae; phylum: Tracheophyta; class: Magnoliopsida; order: Poales ; family: Cyperaceae ; genus: Cyperus ; specificEpithet: rotundus; taxonRank: species; scientificNameAuthorship: L.; nomenclaturalCode: IPNI; taxonomicStatus: Accepted; **Location:** higherGeographyID: SE Europe; higherGeography: SE Europe; continent: Europe; country: Kosovo; countryCode: XK; stateProvince: Gjakovë; county: Gjakovë; municipality: Gjakovë; locality: Radoniq lake; verbatimLocality: Radoniq lake; verbatimElevation: 463 m; locationRemarks: Radoniq Lake – littoral zone, moist sandy–loamy shoreline: 42.477481°N, 20.410191°E, 463 m a.s.l., 12 Aug. 2025. Leg.: Fadil Millaku; verbatimCoordinates: 42.477481°N, 20.410191°E; coordinatePrecision: 2; georeferenceProtocol: GPS; **Identification:** identifiedBy: Fadil Millaku; dateIdentified: 2025; identificationReferences: Kukkonen, I. (1980): Cyperus. — In: Tutin, T.G., Heywood, V.H., Burges, N.A., Moore, D.M., Valentine, D.H., Walters, S.M. & Webb, D.A. (eds.), Flora Europaea, Vol. 5: 282–292. Cambridge University Press, Cambridge.

#### Taxon discussion

This taxon has not previously been reported for the flora of Kosovo, thus representing its first confirmed record in Kosovo. The present record is based on field-collected material that was taxonomically verified using current regional floras and compared with herbarium material from UPH. Its occurrence extends the known distribution range of the species in the central Balkans and contributes to completing the floristic knowledge of Kosovo. No earlier confirmed records exist in the national or regional literature.

#### Notes

The species was recorded along the shores of Radoniq Lake, where it occurred on moist, sandy–loamy substrates within the upper littoral zone (Fig. 1d). At the site, plants were growing in open, periodically exposed shoreline patches, characteristic of fluctuating water levels. Ecologically, *C. rotundus* is a thermophilous species with a broad ecological amplitude, frequently associated with disturbed or seasonally moist habitats. In a wider biogeographical context, it is a pantropical–subtropical taxon that reaches its northernmost European distribution in parts of south-eastern Europe. The occurrence of this species in Kosovo indicates the presence of suitable microhabitat conditions for this warm-adapted sedge along the shores of Radoniq Lake.

## Discussion

**Figure 2.**
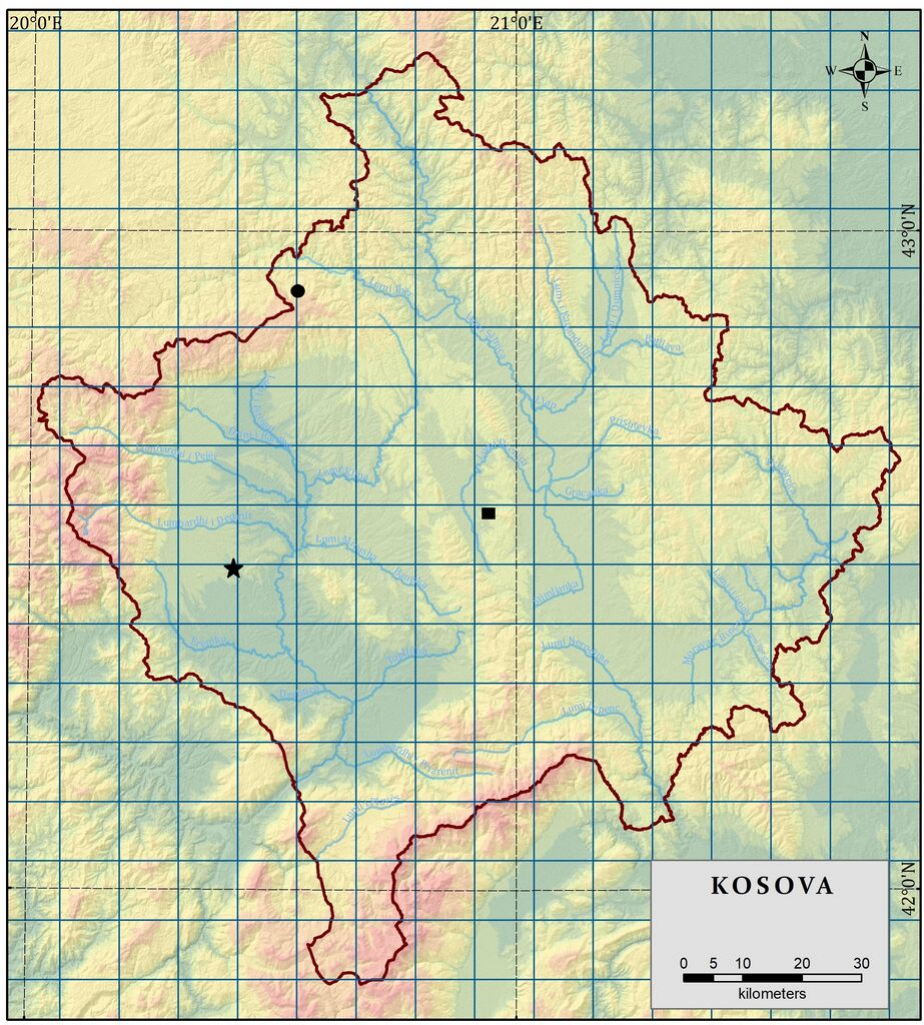
Distribution of the four newly-recorded taxa in Kosovo. The black circle (●) marks the site of Lycopodium annotinum subsp. annotinum on Mokna Mt. (Albanian Alps of Kosovo). The black square (■) indicates the locality of *Cyperus fuscus* L. on Golesh Mt. (Mirenë). The black star (★) denotes Radoniq Lake, where two species, *Cyperus rotundus* L. and *Najas marina* L., were recorded.

Prior to this study, the Lycopodiaceae of Kosovo comprised three confirmed taxa: *Diphasiastrum alpinum* (L.) Holub, *Huperzia selago* (L.) Bernh. ex Schrank & Mart. and Lycopodium clavatum L. subsp. clavatum. Both *D. alpinum* and *H. selago* are of particular conservation interest; they are listed as Endangered (EN) in the Red Book of Vascular Flora of Kosovo and are also considered plant species of international importance for the country (Millaku et al. 2013, Millaku et al. 2025). Their distribution in Kosovo (Fig. 2) is restricted to high-mountain alpine habitats of the Sharr Mountains and the Albanian Alps, where they typically occur in cold, moist microhabitats characteristic of the upper-montane and subalpine zones (Rexhepi 1986, Millaku et al. 2013).

In contrast, Lycopodium clavatum subsp. clavatum is somewhat more widespread, with confirmed occurrences in the Sharr Mountains (Kobilicë, Maja e Zezë) and in the Shalë e Bajgorës Region of the Kopaonik Mountains. Despite this, the family as a whole has remained relatively poorly represented in the national flora and no verified records of Lycopodium annotinum subsp. annotinum existed prior to this study.

The confirmation of L. annotinum subsp. annotinum therefore adds an important fourth member to the Lycopodiaceae of Kosovo and marks the first documented occurrence of this boreal–montane taxon in the country. With this record, the genus *Lycopodium* is now represented by two taxa in Kosovo. The species’ presence in upper-montane spruce forests of Mokna Mt. highlights the existence of suitable microrefugial habitats and further emphasises the botanical significance of the Albanian Alps within Kosovo’s flora (Schönswetter and Schneeweiss 2018, Millaku et al. 2025).

The genus *Najas* has thus far been entirely absent from the documented flora of Kosovo and no verified records or historical mentions exist for it. Despite being widespread across much of Europe and occurring in neighbouring regions, members of the genus are easily overlooked due to their fully submerged habit, delicate morphology and frequent occurrence in shallow water zones that are rarely surveyed in detail. Aquatic macrophytes in general have received comparatively little floristic attention in Kosovo (Bytyqi et al. 2020) and several freshwater systems, slow-flowing streams and marshy lake margins, remain underexplored (Bytyçi et al. 2022).

In this context, the confirmation of *N. marina* represents the first documented occurrence of the genus *Najas* in Kosovo. Its detection along the shores of Radoniq Lake demonstrates that suitable ecological conditions exist in at least some of the country’s lentic habitats to support submerged hydrophytic vegetation. As *N. marina* is a widespread Eurasian species frequently associated with nutrient-rich or moderately alkaline standing waters, its presence is biogeographically plausible; however, the absence of earlier records suggests that aquatic habitats have been systematically overlooked in national floristic research. The addition of Najas marina not only fills a significant gap in the knowledge of Kosovo’s aquatic flora, but also underscores the need for more comprehensive surveys of freshwater ecosystems, which may harbour additional unrecorded taxa.

The confirmation of *Cyperus fuscus* and *C. rotundus* represents a particularly important contribution to the floristic knowledge of Kosovo, as no species of the genus *Cyperus* has ever been verified for the country. Even in the most systematic and comprehensive taxonomic syntheses available for the region, such as the vascular plant checklist of Serbia (Niketić and Tomović 2018), which also includes data for Kosovo, not a single *Cyperus* species was reported from Kosovo, indicating a long-standing gap in the documentation of this genus within the country. Moreover, even a detailed floristic survey of Mt. Golesh itself, where *C. fuscus* has now been recorded, did not previously document the species (Krasniqi et al. 2019), further illustrating how inconspicuous and easily overlooked taxa of this genus can be.

From a broader perspective, the genus *Cyperus* includes ecologically significant taxa that play important roles in wetland dynamics, shoreline stabilisation and the functioning of periodically inundated habitats. Many taxa within the genus are pioneers in fluctuating or ephemeral microhabitats, contributing to early-successional processes. Despite this ecological importance, *Cyperus* species have been under-documented in this part of Europe. Their often small size, ephemeral life cycles and the presence of numerous morphologically similar taxa make the group taxonomically challenging, a factor that has led to widespread under-collection and perhaps misidentification throughout the region.

This pattern is reflected in Kosovo, where the family Cyperaceae as a whole has received only limited research attention. To date, the only genus studied in detail has been *Carex*, which was recently revised and updated for the flora of Kosovo (Berisha et al. In press). In contrast, other cyperaceous genera, *Cyperus* amongst them, have remained largely overlooked, both in historical herbarium material and in modern field surveys.

Against this background, the confirmation of *C. fuscus* and *C. rotundus* as new national records carries notable significance. Together, these findings demonstrate that Kosovo hosts suitable ecological conditions for multiple *Cyperus* species across distinct habitat types.

Taken together, the four newly-confirmed taxa illustrate the ecological breadth and biogeographical complexity of Kosovo’s flora. They originate from markedly different habitat types: boreal-montane coniferous forests (*Lycopodium annotinum*), submerged and littoral vegetation (*Najas marina*), serpentine foothill streams (*Cyperus fuscus*) and warm, periodically exposed lakeshore substrates (*C. rotundus*). Their simultaneous discovery underlines Kosovo’s position as a transitional zone where Alpine, Continental and sub-Mediterranean floristic influences converge (Rexhepi 1997, Berisha et al. 2025a). The confirmation of taxa with such contrasting ecological preferences demonstrates that a diverse set of underexplored habitats persists across relatively small geographical distances and that these environments continue to harbour overlooked or previously undocumented species.

The addition of these four taxa to the national flora reinforces the need for continued systematic surveys in Kosovo, particularly in habitats that have historically received limited attention, such as aquatic systems, ephemeral wetlands, upper-montane coniferous forests and serpentine landscapes. The detection of species previously unrecorded not only fills long-standing gaps in floristic knowledge, but also improves the accuracy of regional distribution maps and future conservation assessments.

### Conclusions

The newly-documented records of four vascular plant taxa (Lycopodium annotinum subsp. annotinum, *Najas marina*, *Cyperus fuscus* and *C. rotundus*) for the flora of Kosovo increase the known diversity of three genera previously unverified in the country and address important gaps in the national floristic inventory. This study documents four vascular plant taxa: Lycopodium annotinum subsp. annotinum, Najas marina, Cyperus fuscus and C. rotundus – as confirmed new records for the flora of Kosovo. The newly-recorded taxa originate from ecologically distinct habitat types. In phytosociological terms and habitat type terms (Habitats Directive 92/43/EEC, Annex I), Lycopodium annotinum L. subsp. annotinum occurs in moist, shaded microhabitats within montane Norway spruce forests (phytosociologically *Vaccinio*-*Piceetea*), corresponding to Annex I habitat 9410. *Najas marina* L. was recorded as a submerged macrophyte in shallow lake-margin waters of Radoniq Lake (vegetation of *Potametea* s.l.), best matching Annex I habitat 3150. Both *Cyperus fuscus* L. (seasonal stream margins) and *C. rotundus* L. (periodically exposed upper littoral lake shore) are linked to pioneer, intermittently wet muddy or sandy bank communities (*Isoëto*-*Nanojuncetea* / muddy-bank vegetation), corresponding primarily to Annex I 3270 and locally to 3130 on drawdown shores where such vegetation develops. This highlights the wide environmental variability within Kosovo and the ongoing under-exploration of several key ecosystems, particularly aquatic habitats. By integrating verified field observations with herbarium documentation, this study contributes to a more comprehensive understanding of Kosovo’s vascular plant diversity and its biogeographical context within the central Balkans. Continued systematic fieldwork, combined with targeted surveys in under-represented habitats and careful taxonomic evaluation, will remain essential for further refining the country’s flora and supporting future conservation and ecological research.

## Supplementary Material

XML Treatment for Lycopodium annotinumannotinum

XML Treatment for Najas marina

XML Treatment for Cyperus fuscus

XML Treatment for Cyperus rotundus

## Figures and Tables

**Figure 1a.**
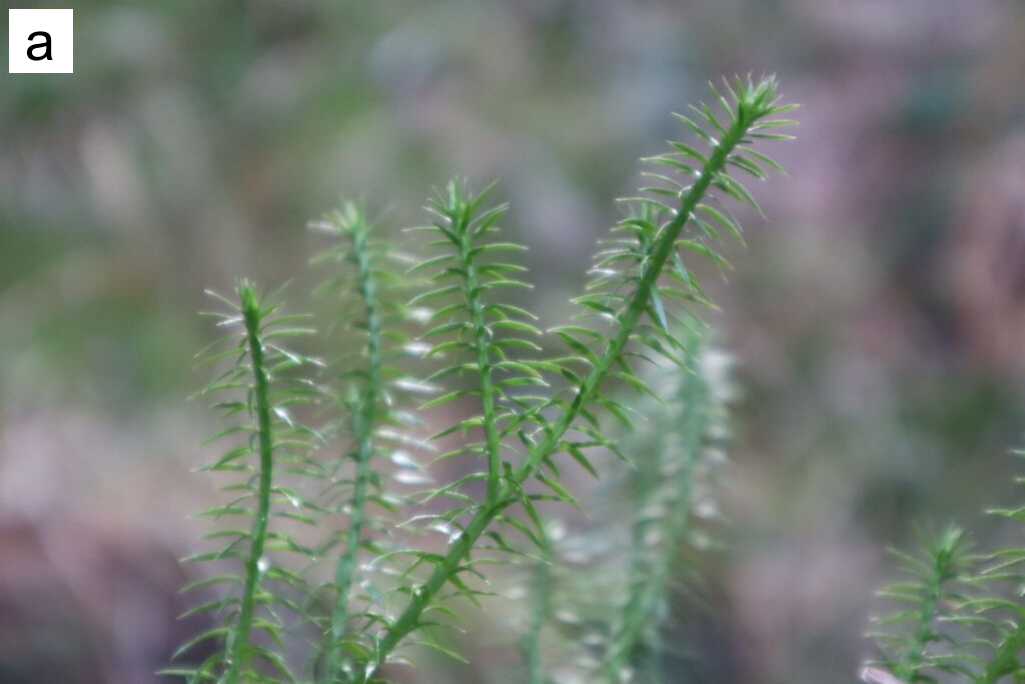
Lycopodium annotinum L. subsp. annotinum;

**Figure 1b.**
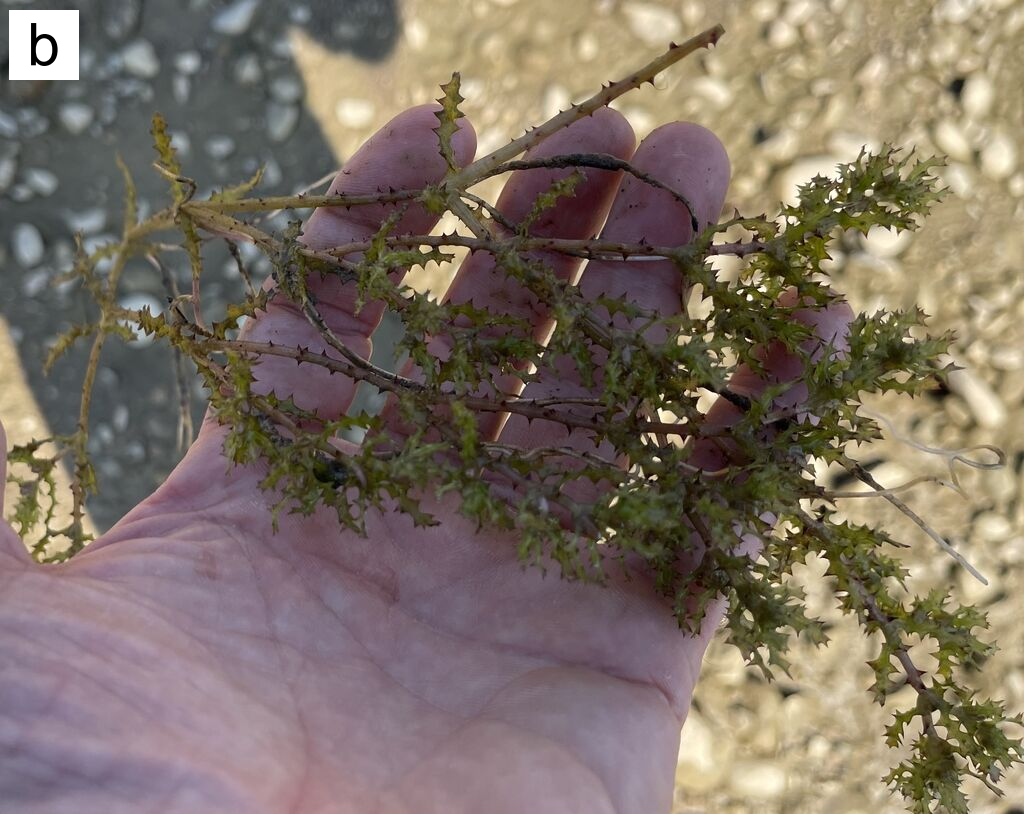
*Najas marina* L.;

**Figure 1c.**
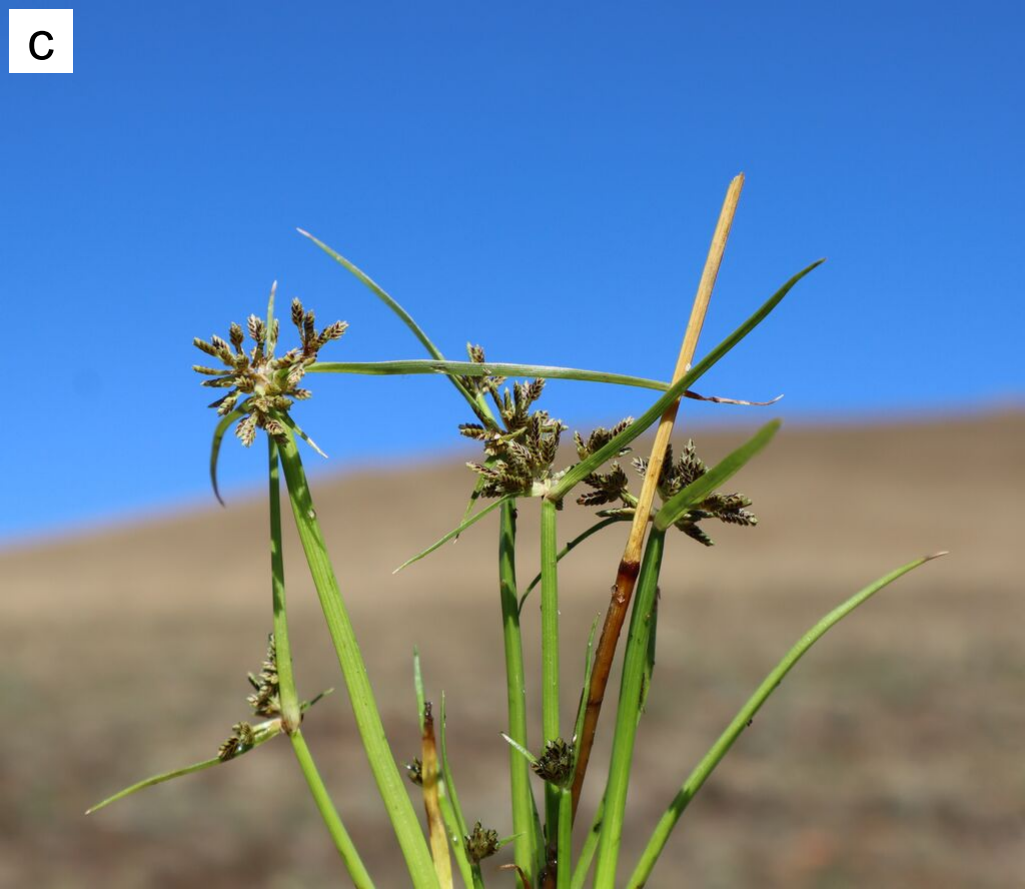
*Cyperus fuscus* L.;

**Figure 1d.**
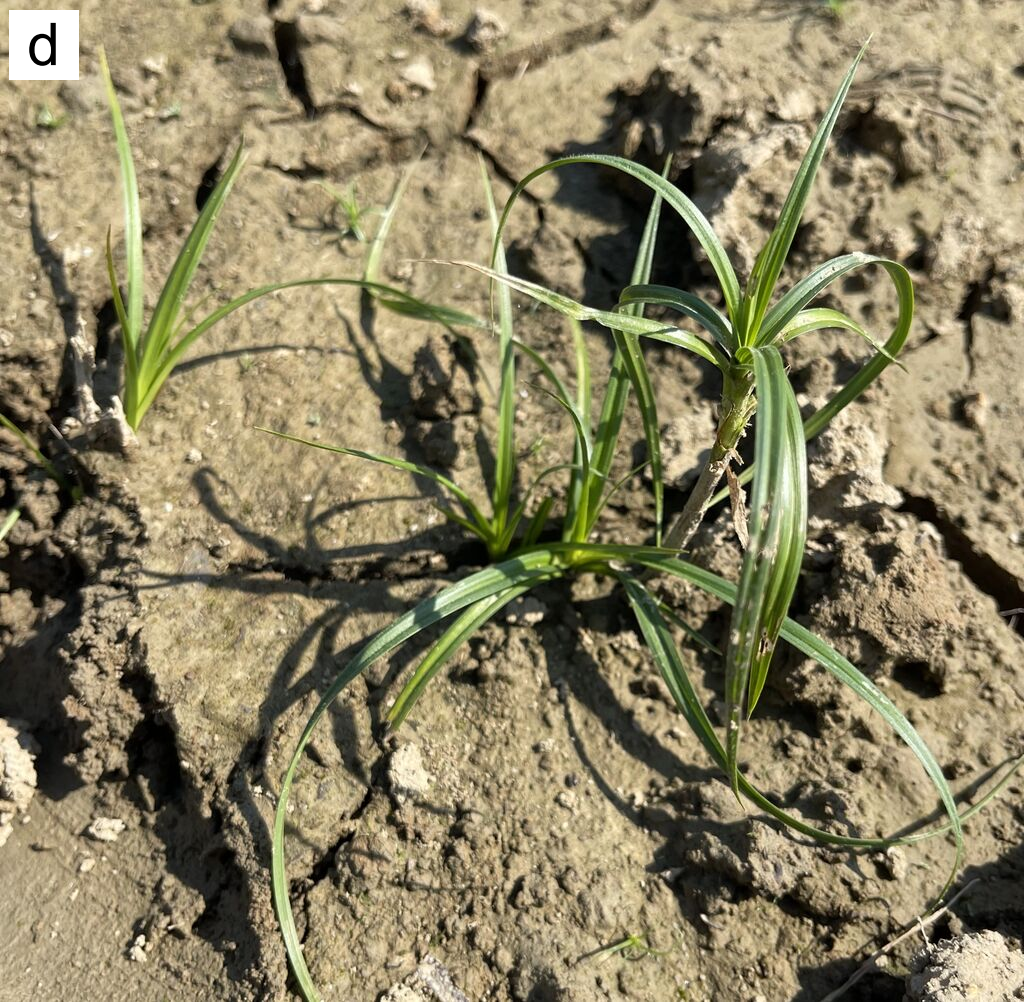
*Cyperus rotundus* L.
